# Association Between Clinical Factors and Result of Immune Checkpoint Inhibitor Related Myasthenia Gravis: A Single Center Experience and Systematic Review

**DOI:** 10.3389/fneur.2022.858628

**Published:** 2022-04-07

**Authors:** Jiayu Shi, Ying Tan, Yangyu Huang, Ke Li, Jingwen Yan, Yuzhou Guan, Li Zhang

**Affiliations:** ^1^Department of Neurology, Peking Union Medical College Hospital, Chinese Academy of Medical Sciences, Beijing, China; ^2^Department of Respiratory and Critical Care, Peking Union Medical College Hospital, Chinese Academy of Medical Sciences, Beijing, China

**Keywords:** myasthenia gravis, MGFA, QMG, immune-related adverse effects, immune checkpoint inhibitors

## Abstract

**Background:**

Neurological immune-related adverse events (nirAEs) are rare toxicities of immune-checkpoint inhibitors (ICI). With the increase use of ICIs, incidence of nirAEs is growing, among which ICI related MG (irMG) is causing high fatality rate. Given the limited evidence, data from a large cohort of patients with irMG is needed to aid in recognition and management of this fatal complication.

**Objective:**

This study aimed to summarize clinical characteristics of irMG and explore predictors of irMG clinical outcome.

**Methods:**

We summarized our institution's patients who were diagnosed as irMG between Sep 2019 and Oct 2021. We systematically reviewed the literature through Oct 2021 to identify all similar reported patients who met inclusion criteria. As the control group, patients with idiopathic MG were used. We collected data on clinical features, management, and outcomes of both irMG and idioMG cases. Further statistical analysis was conducted.

**Results:**

Sixty three irMG patients and 380 idioMG patients were included in the final analysis. For irMG patients, six were from our institution while the rest 57 were from reported cases. The average age of irMG patients is 70.16 years old. Forty three were male. Average time from first ICI injection to symptom onset was 5.500 weeks. Eleven patients had a past history of MG. Higher MGFA classification and higher QMGS rates were observed in irMG patients compared to idioMG patients. For complication, more irMG patients had myositis or myocarditis overlapping compared to idioMG patients. The most commonly used treatment was corticosteroids for both idioMG and irMG. Twenty one patients (35%) with irMG had unfavorable disease outcome. Single variate and multivariate binary logistic regression proved that association with myocarditis, high MGFA classification or QMGS rates at first visit were negatively related to disease outcome in irMG patients.

**Conclusion:**

irMG is a life-threatening adverse event. irMG has unique clinical manifestations and clinical outcome compared to idioMG. When suspicious, early evaluation of MGFA classification, QMGS rates and myositis/myocarditis evaluation are recommended.

## Introduction

Immune checkpoint inhibitors (ICIs) are regarded as effective treatments for different types of advanced cancers (1). Despite impressive benefits observed from using ICIs, these treatments may be associated with serious immune-related adverse events (irAEs) caused by the induction of off-target inflammatory and autoimmune responses (1, 2).

ICI-related neurological adverse events are relatively infrequent; however, pooled analyses have shown that they are associated with increased morbidity and mortality (3–5). Myasthenia gravis (MG) is an autoimmune disorder mediated by autoantibodies, including anti-acetylcholine receptor (AChR) or anti-muscle associated receptor tyrosine kinase (MUSK) antibodies that target the neuromuscular junction (6). MG induced by ICI treatment or ICI-induced relapse of pre-existing MG is known as immune-related MG (irMG) (7). The incidence rate of irMG is 0.1%−0.2% according to the current literature (2, 8). Because of the low incidence rate and limited number of described cases, characterization of clinical features and prediction of disease outcome for irMG is difficult based on a patient's clinical manifestations. In this study, we described the clinical features of 63 patients with irMG and aimed to identify possible factors that may be useful for predicting irMG prognosis.

## Materials and Methods

### Patients

**Figure 1:**
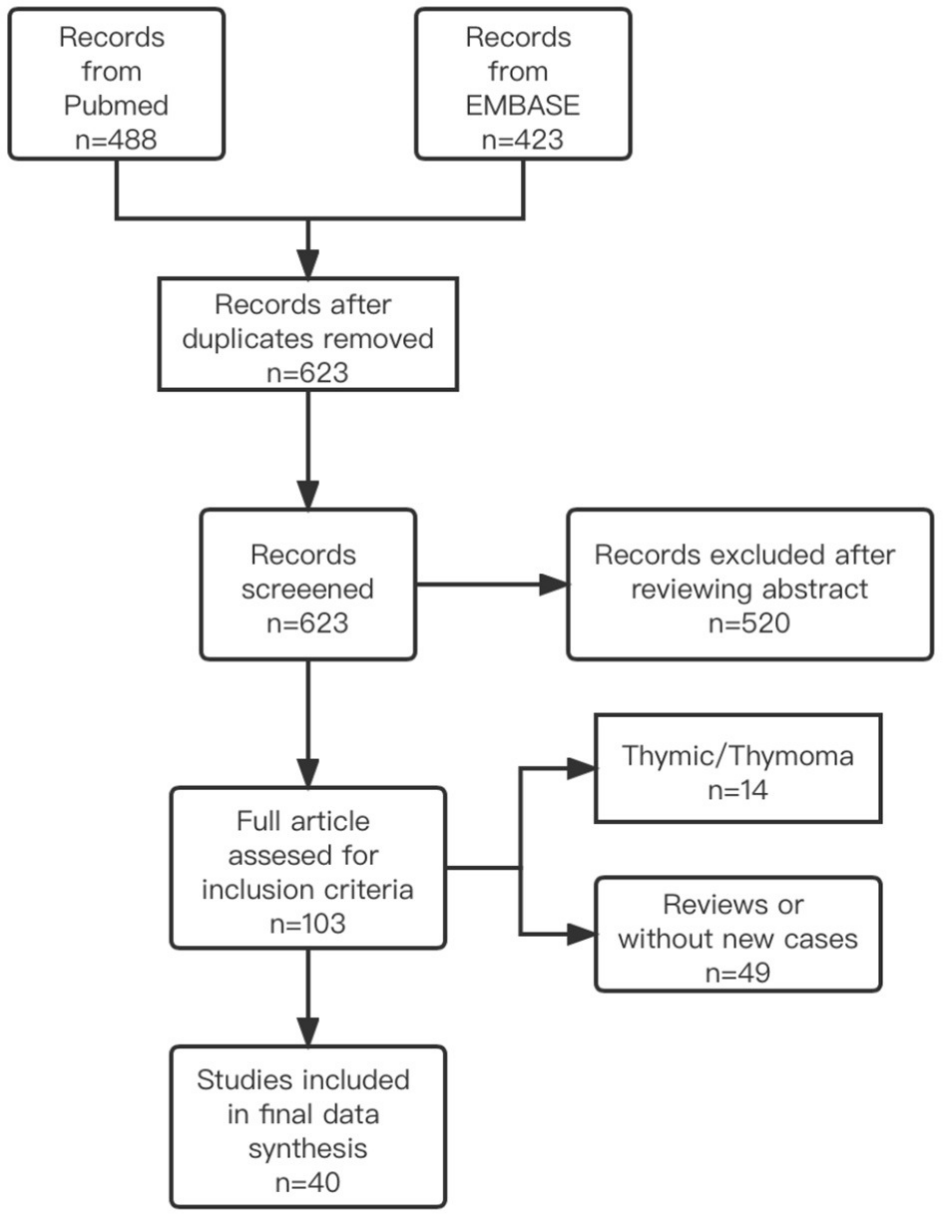
Flow gram describing the systematic search and study selection process.

Patients who were diagnosed with irMG at PUMCH between September 2019 and October 2021 were included in the study cohort. We also searched the PubMed and EMBASE databases through October 2021 for case reports, case series, and observational studies that described patients with cancer and MG who received ICI treatment. The database searches did not include language or study design restrictions. Titles and abstracts were screened by two independent investigators to identify potentially relevant articles. Then, the full text of each selected article was retrieved and reviewed. A detailed clinical description of each patient was generated. The keywords included in the search were (“immune checkpoint inhibitors” OR “nivolumab” OR “ipilimumab” OR “pebrolizumab” OR “avelumab” OR “durvalumab” OR “atezolizumab” OR “anti-PD-1” OR “anti-PD-L1” OR “anti-CTLA-4”) AND (“immune related MG” OR “irMG” OR “MG” OR “Myasthenia Gravis” OR “ocular Myasthenia Gravis”). An inclusion diagram of patients is shown in Figure 1. The quality appraisal of the reported cases from the literature is shown in Supplementary Table 3. The inclusion criteria for both the PUMCH patients and cases identified in the literature included: (1) diagnosed with cancer; (2) any type of ICI used before MG onset or relapse; (3) definite or probable diagnosis of new-onset MG or deterioration of symptoms of well-controlled MG; and (4) a detailed description of the patient's clinical course was available. A definite diagnosis of MG was based on the presentation of ocular and/or systemic muscle weakness and at least one of the following criteria: (1) elevated titers of anti-AChR or anti-MUSK antibodies, (2) findings suggestive of MG in electrodiagnostic tests, (3) positive edrophonium test, or (4) positive ice pack test. A probable diagnosis of MG was made based on high clinical suspicion by the neurologist's report that confirmed the diagnosis of MG. We excluded patients with thymoma as an indicator for ICI treatment.

### Controls

We also studied patients with idiopathic MG (idioMG), who were diagnosed at the PUMCH Neurology Department and registered at PUMCH MG registry as the control group. We have excluded idioMG patients with thymoma.

### Methods

For both the PUMCH and literature identified patients, we extracted variables for patient demographics and baseline characteristics, including age, sex, type of ICI, indication of ICI, cancer staging, time between disease onset and first and last ICI injection, and severity of irAEs. We assessed the clinical severity of irMG using the Myasthenia Gravis Foundation of America (MGFA) classification system. For PUMCH patients, the quantitative myasthenia gravis score (QMGS) was determined by a trained neurologist at each patient's first visit. The QMGS was also collected for literature-identified patients if available. Data on clinical manifestations of MG (ptosis, diplopia, dyspnea, limb weakness and dysphagia); titer results for anti-AchR, anti-MUSK, and anti-titin antibodies; and overlap with myositis, myocarditis, or other system irAEs were also collected if available. Myositis was defined as elevated creatine kinase (CK) levels after disease onset. Myocarditis was defined as elevated cardiac troponin I levels, dynamic changes in electrocardiogram data, or symptoms of acute coronary artery syndrome. An unfavorable outcome was defined as tracheotomy, endotracheal intubation, or death directly caused by ICI-related MG.

### Statistical Analysis

Baseline characteristics of irMG group were evaluated using frequencies and percentages for categorical data, while median and range were used to describe continuous data. Comparisons of categorical variables between control group and patient group were tested for significance using the x2 test. Continuous variables were compared using the Mann-Whitney U test. We performed single variate binary logistic regression analyses to determine the odds ratios (ORs) for associations between certain clinical or demographic factors and risk of unfavorable outcomes for irMG. Factors that were significantly associated with an unfavorable outcome were analyzed together in a multivariate binary logistic regression model. This analysis was performed with the maximal level of adjustment. All tests were 2-sided, and Bonferroni correction was applied to the α level to adjust for multiple comparisons. Bonferroni-adjusted *p* values are reported in the tables. Statistical analyses were carried out using the SPSS 24.0 statistical package (SPSS; Chicago, IL, USA). The study was approved by the local ethics committee.

## Results

For irMG group, six patients from PUMCH were diagnosed with irMG. Of 623 unique articles from the literature, 40 publications describing 57 patients met the inclusion criteria (3, 6, 9–48). Therefore, a total of 63 patients were included in our final analysis. For idioMG group, we included 380 patients from PUMCH MG registry during the same period.

### irMG Patient Demographic and Baseline Characteristics

irMG patients' characteristics are shown in detail in Supplementary Table 1. A summary of demographic and baseline characteristics is shown in Table 1. The most common indication for ICIs was melanoma followed by urethral and lung carcinoma. The majority of patients had progressive tumor staging, and inhibitors of programmed cell death 1 (PD-1) were the most commonly applied therapeutics in the total cohort. The median time from ICI injection to symptom onset was 5 weeks, while the median time from the last ICI injection to symptom onset was 10 days. The severity of irAEs in the majority of patients in our cohort were classified at level IV. More than 60% of the patients had irAEs involving other systems. The most commonly involved system was cardiovascular system, followed by digestive system. Skin and hematological system irAEs were also observed.

**Table 1:** Demographic and baseline characteristics of patients.

	**Total cohort,** **(*n* = 63), *N* (%)**	**PUMCH,** **(*n* = 6), *N* (%)**
Indication for ICI		
Lung Carcinoma	10 (16.1)	
Melanoma	31 (50)	0 (0)
Urethral Carcinoma	13 (21.0)	0 (0)
Gynecological Carcinoma	1 (1.6)	1 (16.7)
Digestive system neoplasm	2 (3.2)	1 (16.7)
Others	3 (8.1)	1 (16.7)
Tumor Staging, *N* (%)
1	0	0
2	2 (4.7)	1 (16.7)
3	7 (16.3)	0 (0)
4	34 (79.1)	5 (83.3)
Type of ICI applied, *N* (%)
PD-1	45 (73.8)	6 (100)
CTLA-4	8 (13.1)	0 (0)
PD-1+CTLA-4	6 (9.8)	0 (0)
Others	2 (3.3)	0 (0)
Time from first ICI injection to symptom onset, median weeks (range)	5 (1–28)	5.5 (2–9)
Time from last ICI injection to symptom onset, median days (range)	10 (1–35)	13.5 (12–28)
Level of irAEs		
I	12 (19.4)	0 (0)
II	10 (16.1)	1 (16.7)
III	12 (19.4)	2 (33.3)
IV	28 (45.2)	3 (50.0)
Complicated with irAEs of other systems, *N* (%)
Myocarditis	21 (33.3)	2 (33.3)
Elevated liver enzymes	7 (11.1)	2 (33.3)
Skin	4 (6.35)	1 (16.7)
Colitis or diarrhea	3 (4.76)	0 (0)
Hematological	2 (3.17)	1 (16.7)
Renal failure	1 (1.59)	0 (0)

### irMG Characteristics and Comparison With idioMG Group

The patients' irMG characteristics, treatments, and outcomes are shown in detail in Supplementary Table 2. A comparison of irMG and idioMG characteristics are shown in Table 2. Among the 63 patients identified with irMG, 11 had a past history of well-managed MG and presented with a flare-up of MG after ICI initiation. The MGFA classification and QMGS rates clearly demonstrated that the disease was more severe in patients with irMG than in patients with idioMG. For clinical manifestations, bulbar symptoms and dyspnea were seen more frequently in patients with irMG. Serologic tests revealed that the frequency rates of anti-AchR antibody and anti-Musk antibody were significantly higher in idioMG group. Besides, the titer of anti-AChR antibodies was relatively low in patients with irMG compared to patients with idioMG. In irMG group, three patients were positive for anti-titin antibodies among nine patients tested (33.3%), which was not commonly seen in idioMG group. In irMG group, markedly elevated CK levels were observed with an average level of 5206.7 IU/L, which was scarcely found in idioMG group. In irMG group, 21 patients were diagnosed with myocarditis, while no patient had cardiac muscle involvement in idioMG group. Sixty one patients (96.8%) from irMG group required hospitalization after disease onset. Corticosteroids were used in more than 90% of patients for management for both irMG and idioMG. IVIg and PLEX were most commonly added to reduce the rapid progression of symptoms in irMG patients while infliximab and rituximab were used in 2 and 1 patients, respectively (33). For idioMG, PLEX was not commonly conducted in our center and we have no experience of using infliximab or rituximab. Unfavorable outcomes including death, intubation or tracheotomy was observed in 21 patients (35%) in irMG group, among which 14 patients (66.7%) died. Reasons of unfavorable outcomes include onset of a myasthenic crisis (13, 62%), infection or other complication (3, 14%) and cardiac incidence (5, 24%). Compared to irMG group, the incidence rate of unfavorable outcomes in idioMG group is relatively low. Discontinuation or withholding of ICI was recommended for 61 patients (97%) in our cohort, while 2 patients continued ICI treatments with well-controlled MG symptoms (41).

**Table 2:** irMG characteristics and treatment.

	**irMG,** **(*n* = 63),** ***N* (%)**	**idioMG,** **(*n* = 380),** ***N* (%)**	***p***
Median age, years (Range)	72 (44–86)	52 (2–84)	0.000
Male, *N* (%)	43 (69.4)	174 (45.8)	0.000
Past history of MG, *N* (%)	11 (19.0)	-	-
MGFA classification at first visit, *N* (%)
I	16 (25.8)	122 (32.1)	0.001
II	10 (16.1)	133 (35.0)	
III	15 (24.2)	84 (22.1)	
IV	21 (34.9)	30 (7.9)	
V	0 (0.0)	11 (2.9)	
Clinical presentation, *N* (%)
Ptosis	49 (89.1)	334 (88.2)	0.821
Diplopia	43 (78.2)	270 (71.3)	0.457
Dyspnea	30 (55.6)	41 (10.8)	0.001
Limb weakness	34 (63.0)	226 (59.6)	0.824
Dysphagia	32 (59.3)	69 (18.2)	0.002
QMGS rates at disease onset, (SD)	18.17 (11.4)	12.32 (8.2)	0.012
Antibody
Positive anti-AchR Ab, *N* (%)	27 (56.3)	277 (73.0)	0.050
Average anti-AchR Ab, nmol/L, (SD)	4.5 (4.1)	7.8 (13.3)	0.081
Positive anti-Musk Ab, *N* (%)	1 (1.6)	27 (7.0)	0.020
Positive anti-Titin Ab, *N* (%)	3 (33.3)	NA	-
Complicated with myositis, *N* (%)	31 (63.3)	32 (8.4)	0.000
Complicated with myocarditis, *N* (%)	21 (41.2)	0 (0)	0.000
CK level, μmol (SD)	5206.7 (5048.3)	137.2 (125.1)	0.000
Treatment, *N* (%)
IVIg	1 (1.9)	5 (1.3)	0.716
IVIg + corticosteroids	11 (20.4)	116 (30.5)	0.213
IVIg + corticosteroids + PLEX	18 (33.3)	10 (2.7)	0.002
Corticosteroids + PLEX	8 (14.8)	7 (1.8)	0.001
Corticosteroids	16 (29.6)	201 (52.9)	0.137
Infliximab	2 (3.2)	0 (0)	0.145
Rituximab	1 (1.6)	0 (0)	0.219
Outcome	
Tracheotomy, Intubation, or Death	21 (35.0)	23 (6.1)	0.001
Improvement	40 (65.0)	-	

### Associations Between Demographic and Clinical Factors and irMG Outcome

**Figure 2:**
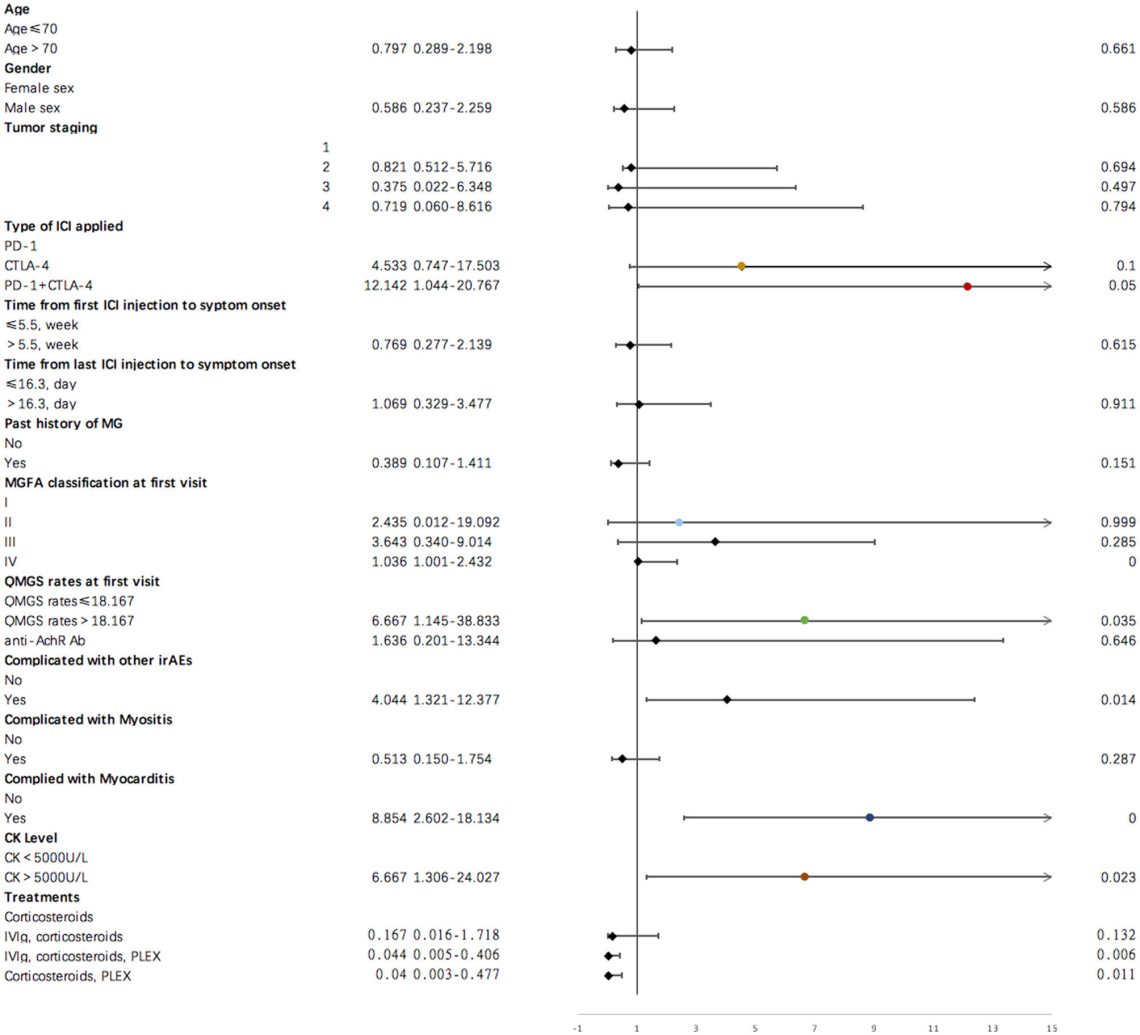
Results of single variate binary logistic regression for demographic and clinical factors and irMG outcome.

**Figure 3:**
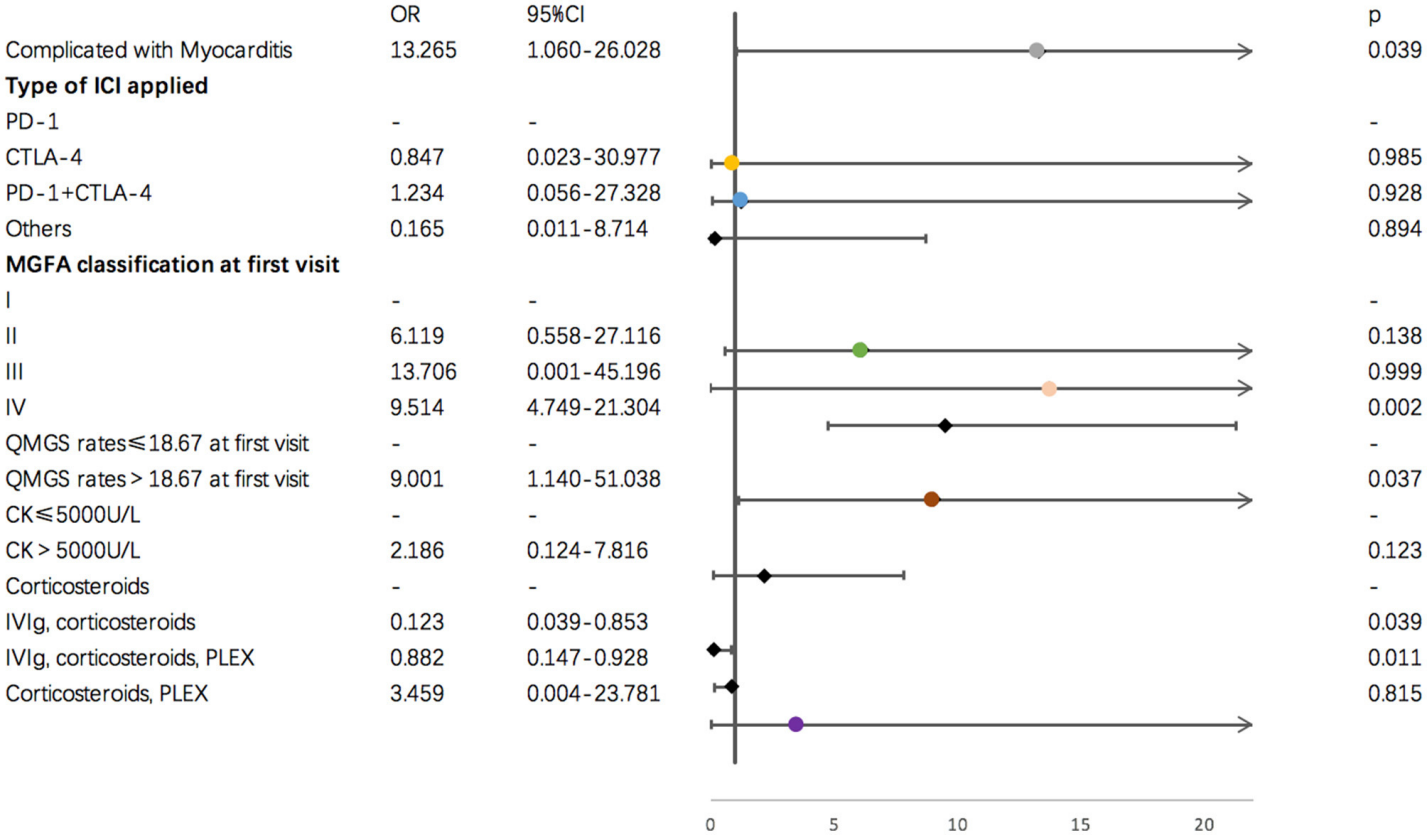
Results of multi-variate binary logistic regression for demographic and clinical factors and irMG outcome.

Results of single variate binary logistic regression are shown in Figure 2. Application of cytotoxic T-lymphocyte associated protein 4 (CTLA-4) and PD-1 inhibitors together was negatively related to an unfavorable outcome for ICI-related MG (*OR* = 12.142, *p* = 0.050). Evaluation parameters for MG severity, which included QMGS and MGFA classification at the first clinical visit, were indicative of disease outcome. A QMGS > 18.167 (*OR* = 6.667, *p* = 0.035) and MGFA classification IV (*OR* = 1.036, *p* = 0.000) were both related to unfavorable disease outcome. An overlap of myocarditis with irAEs in other systems was significantly associated with unfavorable irMG outcome. Although associated with myositis was not relevant to disease outcome, creatine kinase levels > 5,000 U/L were negatively related to disease outcome (*OR* = 6.667, *p* = 0.023). For treatment, we found that, compared to using steroids alone, administering IVIg, steroids plus PLEX, or IVIg plus steroids may be protective factors for irMG outcome. From the factors that were analyzed in the single variate binary logistic model, we included the type of ICI applied, MGFA classification, QMGS, overlap of myocarditis with other system irAEs, and treatments in the multivariate binary logistic regression model. The multivariate analysis (Figure 3) showed that, associated with myocarditis, QMGS ≥ 18.167 and MGFA classification IV were negatively related to the outcome of irMG. Compared with corticosteroids alone, utilization of IVIg and PLEX may be a positive prognostic factor for irMG.

## Discussion

In this study, we report an extensive case series of ICI-related MG with detailed clinical features, treatments, and disease outcome. Our study innovatively identified several clinical factors that may be useful for predicting irMG prognosis.

Our findings support that irMG has several different clinical features compared with idioMG, which has also been proved by previous studies (10, 11). Demographically, the age at diagnosis of irMG was significantly greater than that of patients with idioMG. For clinical severity, the majority of idioMG patients fall within the MGFA classes I and II at the time of diagnosis and present with a slow clinical deterioration course (47), while the majority of irMG patients were categorized in MGFA classes III and IV at the first visit with a high QMGS rates. Serologically, the positive rate of anti-AchR antibody in idioMG patients has been reported to be around 70–80% (49–51), which is statistically higher than the positive rate of irMG group. For anti-MUSK antibody, positive rate in idioMG patients is ~5–10% (51), while in irMG group, positive rate was only 1.6%. This finding shows that the prevalence of seronegative patients in irMG was higher than that in classical MG, which has been previously proved by other studies (7, 41, 52). The demographic and serological differences could be caused by the bias due to that case reports and case series tend to report irMG patients with more severe clinical manifestations, still the differences that we observed indicated that irMG and idioMG are clinically distinct disease entity.

We also observed that irMG were more likely to be associated with myositis or myocarditis, which has been described in only 0.9% of patients with idioMG (6, 7, 53). Some researchers believe that the elevation of serum CK in patients with irMG reflects inflammatory involvement of skeletal muscles rather than rhabdomyolysis (7). Other investigators have hypothesized that myositis is the main clinical manifestation of irMG patients, whereas a positive antibody result is a marker of activated autoimmunity (23, 54). High association rate of myocarditis in irMG group is noticeable. Although the mechanism of this phenomenon is now still not well-established, molecular mimicry and the critical role of PD-1 signaling pathways in regulating autoimmune responses myocardium might be responsible (19).

It is important for physicians to identify factors that might be indicative of disease outcome. We found that higher MGFA classification and a higher QMGS at the first visit were predictive for an unfavorable disease outcome. Both MGFA classification and QMGS are parameters for prognosis prediction and severity evaluation in idioMG patients, and our study supported that the utilization of these measures is valid in irMG patients. It has been demonstrated that associated with myositis may increase muscle weakness in patients with irMG (7, 51, 53), suggesting that a substantial proportion of an irMG patient's clinical symptoms is associated with the accompanying myositis. Although associating with myositis was not related to disease outcome in our analysis, still in single variate analysis, we have found that CK>5,000 U/L was a negative prognosis factor. Thus, awareness of early recognition of muscle involvement in possible irMG patients is important. In this regard, serum CK tests before and after treatment with irMG is required. Association with myocarditis has been reported to be negatively related to irAEs outcome (22, 47, 54). Because it is not uncommon for irMG patients to have myocarditis [39.7% in our cohort, 20%−40% in published series cases (4, 55, 56)], we believe that particular attention including ECG, echocardiography and serum troponin tests should be conducted for myocarditis identification to allow timely multidisciplinary management.

Our data suggest that patients who received IVIg or PLEX experienced improved irMG outcomes compared with those who received steroids alone. Although corticosteroids are recommended as a first-line treatment for irMG (2, 57), the use of steroids as a sole first-line therapy may not be ideal because steroid use itself can cause an acute exacerbation of MG symptoms (58). Although the worsening in symptoms has been described as transient (59), the use of steroids alone in irMG may be associated with a poorer prognosis because these patients may not be able to survive a transient worsening of symptoms considering their older age and advanced stage of malignancy (41). Apart from this, the role of steroids in controlling immune dysregulation in irMG patients might be limited by the constant presence of the circulating ICIs as the original trigger of irAEs. Since IVIg and PLEX could accelerate the process of ICIs mAbs elimination, they could mediate a faster improvement of symptoms (41). From our clinical experience and analysis, the use of IVIg and PLEX together with corticosteroids has led to favorable outcomes in irMG patients, which has been demonstrated in other studies as well (3, 19, 41, 60). However, for irMG initial treatment, no consistent conclusion could be drawn from the big variety of published reports. Given the small number of patients and the retrospective nature of our study, we think further researches for irMG treatment is highly required. Besides, physicians should be aware of early treatments of vital organ dysfunction. Timely intubation for respiratory failure, pacemaker implantation for fatal arrhythmia, and vasopressor and even extracorporeal membrane oxygenation for cardiogenic shock should be considered if clinically needed and available.

Our study has some limitations. Although it is the first study that identifies possible factors responsible for irMG outcome, the relatively small sample size and retrospective nature of the study design limit the reliability of our study results. Because of the variability in the data available from case reports or case series, there were missing data regarding clinical features, hospital course, and outcomes of some patients, which subjected our results to reporting bias. Besides, the information obtained from the collected case reports represents only a small fraction of the actual number of cases worldwide. Nevertheless, our study enhances the understanding of irMG clinical manifestations and factors involved in irMG prognosis.

With the boost of ICIs utilization and awareness of the disease, we believe that the number of patients with irMG is poised to rapid increase. Additional therapeutic studies concerning irMG in the future are needed to decrease the irAE-related mortality and increase the safety of immune therapy.

## Data Availability Statement

The original contributions presented in the study are included in the article/Supplementary Material, further inquiries can be directed to the corresponding author.

## Ethics Statement

The studies involving human participants were reviewed and approved by Peking Union Medical College Hospital. The patients/participants provided their written informed consent to participate in this study.

## Author Contributions

JS and YG contributed to the conception and design of the study. JS, YT, YH, KL, and JY collected the clinical data. JS and YH contributed to the potential article identifications and article quality evaluation. JS wrote the manuscript. YG and LZ edited the manuscript. All authors read and approved the final manuscript.

## Funding

This work was supported by the National Clinical Cohort Study of Rare Diseases for Key R&D Program (Grant Number: 2016YFC091501) and Beijing Municipal Science & Technology Commission Capital Characteristic Clinic Project Z181100001718145.

## Conflict of Interest

The authors declare that the research was conducted in the absence of any commercial or financial relationships that could be construed as a potential conflict of interest.

## Publisher's Note

All claims expressed in this article are solely those of the authors and do not necessarily represent those of their affiliated organizations, or those of the publisher, the editors and the reviewers. Any product that may be evaluated in this article, or claim that may be made by its manufacturer, is not guaranteed or endorsed by the publisher.
