# Supplementary material for: Association Between Clinical Factors and Result of Immune Checkpoint Inhibitor Related Myasthenia Gravis: A Single Center Experience and Systematic Review
Source: Front Neurol. 2022 Apr 7;13:858628. doi: 10.3389/fneur.2022.858628 (PMC9022009; doi:10.3389/fneur.2022.858628)
Supplement: Supplementary file 1 [file Table_1.DOCX]

Supplementary Table 1. Demographic, oncological characteristics, in the 63 patients included in the cohort with neurological toxicities from ICI treatment.

| Patient ID | Age | Gender | Patient's Origin | Type of Cancer | Tumor Staging | ICI applied | Weeks from first injection to onset | Days from last injection to onset |
| --- | --- | --- | --- | --- | --- | --- | --- | --- |
|  |  | 1 Male, 2 Female |  | 1Lung Carcinoma, 2Melanoma, 3Urological Carcinoma,4Gynecologic Carcinoma,5Gastrointestinal tumors, 6Others |  | 1PD-1, 2CTLA-4, 3CTLA-4+PD1, 4PDL-1 |  |  |
| 1 | 56 | 1 | PUMCH | 6 | 4 | 1 | 9 | 14 |
| 2 | 62 | 1 | PUMCH | 6 | 4 | 1 | 6 | 28 |
| 3 | 51 | 1 | PUMCH | 1 | 4 | 1 | 2 | 13 |
| 4 | 69 | 1 | PUMCH | 1 | 4 | 1 | 3 | 21 |
| 5 | 56 | 1 | PUMCH | 1 | 4 | 1 | 8 | 12 |
| 6 | 69 | 2 | PUMCH | 4 | 2 | 1 | 5 | 10 |
| 7 | 73 | 1 | Reported Cases | 2 | 4 | 1 | 3 | 13 |
| 8 | 84 | 1 | Reported Cases | 2 | 4 | 1 | 12 | 10 |
| 9 | 34 | 2 | Reported Cases | 6 | 3 | 1 | 28 | 12 |
| 10 | 71 | 1 | Reported Cases | 2 | 4 | 2 | 4 | 30 |
| 11 | 71 | 1 | Reported Cases | 3 | N/A | 1 | 4 | 3 |
| 12 | 44 | 1 | Reported Cases | 1 | N/A | 1 | 15 | 1 |
| 13 | 76 | 1 | Reported Cases | 2 | N/A | 1 | 9 | 35 |
| 14 | 74 | 1 | Reported Cases | 2 | N/A | 1 | 3 | 20 |
| 15 | 75 | 1 | Reported Cases | 3 | N/A | 3 | 3 | 26 |
| 16 | 69 | 2 | Reported Cases | 1 | 4 | 1 | 9 | 7 |
| 17 | 69 | 2 | Reported Cases | 2 | 4 | 2 | 6 | 2 |
| 18 | 73 | 2 | Reported Cases | 2 | 3 |  | 7 | 10 |
| 19 | 70 | 2 | Reported Cases | 2 | 4 | 2 | 4 | 8 |
| 20 | 73 | 1 | Reported Cases | 2 |  | 1 | 4 | 4 |
| 21 | 63 | 1 | Reported Cases | 2 | 4 | 1 | 2 | 14 |
| 22 | 68 | 1 | Reported Cases | 2 | 4 | 3 | 1 | 7 |
| 23 | 73 | 1 | Reported Cases | 3 | 2 | 1 | 3 | 4 |
| 24 | 74 | 1 | Reported Cases | 2 | 4 | 2 | 9 | 1 |
| 25 | 73 | 1 | Reported Cases | 1 | 4 | 1 | 3 | 2 |
| 26 | 75 | 1 | Reported Cases | 2 | 4 | 4 | 6 | 3 |
| 27 | 81 | 2 | Reported Cases | 3 | 4 | 4 | 12 | 25 |
| 28 | 61 | 2 | Reported Cases | 3 | 3 | 3 | 3 | 17 |
| 29 | 80 | 1 | Reported Cases | 3 | 4 | 3 | 6 | 2 |
| 30 | 80 | 2 | Reported Cases | 3 | 4 | 3 | 12 | 4 |
| 31 | 74 | 1 | Reported Cases | 2 | 4 | 1 | 12 | 22 |
| 32 | 70 | 1 | Reported Cases | 3 | 4 | 1 | 3 | 11 |
| 33 | 70 | 1 | Reported Cases | 5 | 4 | 1 | 3 | 10 |
| 34 | 83 | 1 | Reported Cases | 2 | 3 | 1 | 5 | 25 |
| 35 | 77 | 1 | Reported Cases | 3 | 4 | 1 | 3 | 20 |
| 36 | 63 | 1 | Reported Cases | 3 | 4 | 1 |  | 34 |
| 37 | 82 | 1 | Reported Cases | 1 | 3 | 1 | 9 | 22 |
| 38 | 57 | 1 | Reported Cases | 2 | 4 | 3 | 7 | 7 |
| 39 | 62 | 2 | Reported Cases | 1 | 4 | 1 | 4 | 14 |
| 40 | 66 | 2 | Reported Cases | 5 | 3 | 1 | 5 | 14 |
| 41 | 59 | 2 | Reported Cases | 2 | 3 | 1 | 10 | 5 |
| 42 | 76 | 1 | Reported Cases | 2 | 4 | 1 | 4 | 1 |
| 43 | 74 | 2 | Reported Cases | 2 | N/A | 1 | 4 | N/A |
| 44 | 53 | 1 | Reported Cases | 2 | N/A | 1 | 6 | N/A |
| 45 | 81 | 1 | Reported Cases | 2 | N/A | 1 | 8.28 | N/A |
| 46 | 85 | 2 | Reported Cases | 2 | N/A | 1 | 3 | N/A |
| 47 | 68 | 1 | Reported Cases | 2 | N/A | 1 | 5 | N/A |
| 48 | 65 | 1 | Reported Cases | 2 | N/A | 1 | 3.42 | N/A |
| 49 | 57 | 1 | Reported Cases | 1 | N/A | 1 | 8.3 | N/A |
| 50 | 73 | 1 | Reported Cases | 3 | N/A | 1 | 2 | N/A |
| 51 | 70 | 2 | Reported Cases | 2 | N/A | 2 | 4 | N/A |
| 52 | 73 | 2 | Reported Cases | 2 | N/A | 2 | 3 | N/A |
| 53 | 72 | 2 | Reported Cases | 2 | N/A | 2 | 7.14 | N/A |
| 54 | 60 | 1 | Reported Cases | 2 | N/A | 2 | 2 | N/A |
| 55 | 86 | 1 | Reported Cases | 6 | N/A | 1 | 3 | 5 |
| 56 | 72 | 1 | Reported Cases | 3 | 4 | 1 | 9 | 3 |
| 57 | 83 | 1 | Reported Cases | 1 | 4 | 1 | 9.5 | 7 |
| 58 | 84 | 2 | Reported Cases | 3 | 4 | 1 | 4 | 3 |
| 59 | 75 | 1 | Reported Cases | 2 | 4 | 1 | 5 | N/A |
| 60 | 81 | 1 | Reported Cases | 2 | 4 | 1 | 12 | 5 |
| 61 | 86 | 2 | Reported Cases | 2 | 4 | 1 | 9 | 20 |
| 62 | 66 | 1 | Reported Cases | 6 | 4 | 1 | 6 | 10 |
| 63 | 56 | 1 | Reported Cases | 6 | 4 | 1 | 3.5 | 1 |
