# Supplementary material for: Association Between Clinical Factors and Result of Immune Checkpoint Inhibitor Related Myasthenia Gravis: A Single Center Experience and Systematic Review
Source: Front Neurol. 2022 Apr 7;13:858628. doi: 10.3389/fneur.2022.858628 (PMC9022009; doi:10.3389/fneur.2022.858628)
Supplement: Supplementary file 2 [file Table_2.DOCX]

Supplementary Table 2. irMG clinical manifestations, blood CK level, antibody results, treatment and outcomes.

| Patient ID | Preexisting MG | MGFA Classification | QMGS rates | Ball muscles and respiratory muscles involvement predominant | Ptosis | Diplopia | Limb weakness | Dysphagia | Dyspnea | Anti-AchR Ab | Anti-Musk Ab | Anti-Titin Ab | CK level (U/L) | Myositis | Myocarditis | Treatment | Outcome |
| --- | --- | --- | --- | --- | --- | --- | --- | --- | --- | --- | --- | --- | --- | --- | --- | --- | --- |
|  | 1 Yes, 2 No |  |  | 1 Yes, 2 No | 1 Yes, 2 No | 1 Yes, 2 No | 1 Yes, 2 No | 1 Yes, 2 No | 1 Yes, 2 No | 1 Positve, 2 Negative | 1 Positve, 2 Negative | 1 Positve, 2 Negative |  | 1 Positve, 2 Negative | 1 Positve, 2 Negative | 1 IVIg, 2IVIg+Steroids, 3IVIg+Steroids+PLEX, 4PELX+Steroids, 5 Steroids, 6PLEX 7 Others | 1 Death caused by irMG, Tracheotomy, Mechanical Ventilation 2 No ventilation needed |
| 1 | 2 | 5 | 38 | 1 | 1 | 1 | 1 | 1 | 1 | 1 | 2 | N/A | 11627 | 1 | 1 | 3 | 1 |
| 2 | 2 | 3 | 18 | 1 | 1 | 1 | 1 | 1 | 1 | 2 | 2 | N/A | 8156 | 1 | 1 | 2 | 2 |
| 3 | 1 | 3 | 16 | 1 | 1 | 1 | 2 | 1 | 1 | 1 | 2 | N/A | 987 | 1 | 1 | 2 | 2 |
| 4 | 2 | 1 | 3 | 2 | 1 | 2 | 2 | 2 | 2 | N/A | 2 | N/A | 2081 | 1 | 1 | 3 | 1 |
| 5 | 2 | 3 | 20 | 1 | 1 | 1 | 1 | 1 | 1 | 1 | 2 | N/A | N/A | 2 | 2 | 2 | 2 |
| 6 | 2 | 2 | 14 | 2 | 1 | 1 | 1 | 1 | 1 | 2 | 2 | N/A | 1560 | 1 | 2 | 2 | 2 |
| 7 | 2 | 2 | 5 | 1 | 1 | 2 | 2 | 2 | 1 | 1 | 2 | N/A | N/A | 2 | 2 | 3 | 2 |
| 8 | 2 | 3 | 17 | 1 | 1 | 1 | 1 | 1 | 1 | 1 | 2 | N/A | N/A | 2 | 2 | 2 | 1 |
| 9 | 1 | 4 | 28 | 1 | 1 | 1 | 2 | 1 | 1 | 2 | N/A | N/A | N/A | 2 | 1 | 2 | 1 |
| 10 | 1 | 5 | N/A | 1 | 2 | 2 | 1 | 1 | 1 | 1 | N/A | N/A | N/A | 2 | 2 | 3 | 1 |
| 11 | 2 | 4 | N/A | 1 | 1 | 2 | 1 | 1 | 1 | 2 | N/A | N/A | 10893 | 1 | 1 | N/A | 1 |
| 12 | 2 | 4 | N/A | 2 | 2 | 2 | 1 | 2 | 2 | N/A | N/A | N/A | 22000 | 1 | 2 | N/A | 2 |
| 13 | 2 | 3 | N/A | 2 | 1 | 1 | 1 | 1 | 2 | 2 | N/A | N/A | 3145 | 1 | 1 | N/A | 2 |
| 14 | 2 | 3 | N/A | 1 | 1 | 1 | 1 | 2 | 2 | 1 | 2 | N/A | 716 | 1 | 2 | N/A | 2 |
| 15 | 2 | 1 | N/A | 2 | 1 | 1 | 2 | 2 | 2 | 1 | 2 | N/A | 200 | 2 | 2 | N/A | 2 |
| 16 | 2 | 3 | N/A | 1 | 1 | 1 | 1 | 2 | 1 | 1 | N/A | N/A | 1156 | 1 | 1 | 1 | 2 |
| 17 | 2 | 3 | 7 | 2 | 1 | 1 | 1 | 1 | 1 | 1 | 2 | N/A | N/A | 2 | 2 | 4 | 2 |
| 18 | 2 | 4 | 12 | 1 | 2 | 2 | 1 | 1 | 1 | 1 | 2 | N/A | N/A | 2 | 2 | 5 | 1 |
| 19 | 2 | 4 | 29 | 1 | 1 | 1 | 1 | 1 | 1 | 1 | 2 | N/A | 1200 | 1 | 2 | 3 | 2 |
| 20 | 2 | 2 | 8 | 2 | 1 | 1 | 1 | 1 | 2 | 1 | 2 | N/A | N/A | 2 | 2 | 2 |  |
| 21 | 1 | 1 | 34 | 1 | 1 | 1 | 1 | 1 | 1 | 1 | N/A | N/A | 10480 | 1 | 2 | 3 | 1 |
| 22 | 2 | 4 | 32 | 1 | 1 | 1 | 1 | 1 | 1 | 1 | N/A | N/A | 5400 | 1 | 1 | 4 | 1 |
| 23 | 2 | 4 | 30 | 1 | 1 | 1 | 1 | 1 | 1 | 1 | 2 | N/A | 8950 | 1 | 1 | 3 | 1 |
| 24 | 2 | 2 | N/A | 2 | 2 | 1 | 2 | 2 | 2 | 2 | N/A | N/A | N/A | 2 | 2 | 5 | 2 |
| 25 | 1 | 1 | 6 | 2 | 1 | 1 | 2 | 2 | 2 | N/A | N/A | 1 | 7311 | 1 | 2 | 5 | 2 |
| 26 | 1 | 4 | 34 | 1 | 1 | 1 | 1 | 1 | 1 | 1 | 2 | 2 | N/A | 2 | 2 | 3 | 1 |
| 27 |  | 4 | N/A | 1 | N/A | N/A | N/A | N/A | N/A | 2 | 1 |  | N/A | 1 | 2 | 4 | 1 |
| 28 |  | 4 | N/A | 1 | N/A | N/A | N/A | N/A | N/A | N/A | N/A | N/A | N/A | 1 | 1 | 2 | 1 |
| 29 |  | 4 | N/A | 1 | N/A | N/A | N/A | N/A | N/A | N/A | N/A | N/A | N/A | 1 | 1 | 4 | 1 |
| 30 | 1 | 4 | N/A | 1 | N/A | N/A | N/A | N/A | N/A | N/A | N/A | N/A | N/A | N/A | 1 | 4 | 1 |
| 31 | 2 | 4 | N/A | 1 | N/A | N/A | N/A | N/A | N/A | N/A | N/A | N/A | N/A | N/A | 1 | 3 | 1 |
| 32 | 2 | 4 | N/A | 1 | N/A | N/A | N/A | N/A | N/A | N/A | N/A | N/A | N/A | 1 | 1 | 4 | 1 |
| 33 | 2 | 4 | N/A | 1 | N/A | N/A | N/A | N/A | N/A | 2 | 2 | 2 | N/A | N/A | 1 | 3 | 1 |
| 34 | 2 | 3 | N/A | 1 | 1 | 1 | 1 | 1 | 1 | 1 | 2 | 1 | 5567 | 1 | 1 | 3 | 2 |
| 35 | 2 | 4 | N/A | 1 | 1 | 1 | 1 | 1 | 1 | 1 | 2 | N/A | 8574 | 1 | 1 | 3 | 1 |
| 36 | 2 | 1 | N/A | 2 | 1 | 1 | 2 | 2 | 2 | 2 | 2 | 2 | 3385 | 1 | 1 | 5 | 2 |
| 37 | 2 | 1 | N/A | 2 | 1 | 1 | 2 | 2 | 2 | 1 | N/A | N/A | N/A | 2 | 2 | 5 | 2 |
| 38 | 2 | 1 | N/A | 2 | 1 | 1 | 2 | 2 | 1 | 2 | 2 | 2 | N/A | 2 | 2 | N/A | 2 |
| 39 | 2 | 1 | N/A | 2 | 1 | 1 | N/A | N/A | N/A | 2 | 2 | 2 | N/A | 2 | 2 | N/A | 2 |
| 40 | 1 | 4 | N/A | 2 | 1 | 1 | 1 | 1 | 2 | N/A | N/A | N/A | 1700 | 1 | 1 | 2 | 1 |
| 41 | 1 | 3 | N/A | 1 | 2 | 2 | 1 | 1 | 2 | 2 | N/A | 2 | N/A | N/A | N/A | 3 | 2 |
| 42 |  | 3 | N/A | 1 | 1 | 1 | 1 | 1 | 1 | N/A | N/A | N/A | N/A | N/A | N/A | 3 | 2 |
| 43 | 2 | 1 | N/A | 2 | 1 | 1 | 2 | 2 | 2 | 1 | N/A | N/A | 654 | 1 | 2 | 5 | 2 |
| 44 | 1 | 1 | N/A | 2 | 1 | 1 | 2 | 2 | 2 | N/A | N/A | N/A | N/A | N/A | N/A | 5 | 2 |
| 45 | 2 | 1 | N/A | 2 | 1 | 2 | 2 | 2 | 2 | N/A | N/A | N/A | N/A | N/A | N/A | 5 | 2 |
| 46 | 2 | 1 | N/A | 2 | 1 | 2 | 2 | 2 | 2 | N/A | N/A | N/A | N/A | N/A | N/A | 5 | 2 |
| 47 | 2 | 1 | N/A | 2 | 1 | 1 | 2 | 2 | 2 | N/A | N/A | N/A | N/A | N/A | N/A | 5 | 2 |
| 48 | 2 | 1 | N/A | 2 | 1 | 1 | 2 | 2 | 2 | N/A | N/A | N/A | N/A | N/A | N/A | 5 | 2 |
| 49 | 2 | 1 | N/A | 2 | 1 | 2 | 2 | 2 | 2 | N/A | N/A | N/A | N/A | N/A | N/A | 5 | 2 |
| 50 | 2 | 2 | N/A | 2 | 1 | 1 | 2 | 2 | 2 | N/A | N/A | N/A | N/A | 1 | N/A | N/A | N/A |
| 51 | 2 | 2 | N/A | 2 | 1 | 1 | 1 | 1 | 2 | 1 | N/A | N/A | 1200 | 1 | 2 | 4 | 2 |
| 52 | 2 | 2 | N/A | 1 | 2 | 2 | 1 | 2 | 1 | 1 | N/A | N/A | N/A | 2 | 2 | 5 | 2 |
| 53 | 2 | 2 | N/A | 2 | 1 | 1 | 1 | 1 | 1 | 1 | N/A | N/A | 1389 | 1 | 2 | 4 | 2 |
| 54 | 2 | 3 | N/A | 2 | 1 | 1 | 1 | 1 | 1 | 1 | N/A | N/A | 10644 | 1 | 2 | 3 | 2 |
| 55 | 2 | 4 | 30 | 1 | 1 | 1 | 1 | 1 | 1 | 1 | 2 | 2 | 6407 | 1 | 1 | 3 | 1 |
| 56 | 2 | 3 | N/A | 1 | 1 | 1 | 1 | 1 | 1 | 2 | 2 | 2 | N/A | 2 | 2 | 2 | 2 |
| 57 | 2 | 2 | 14 | 2 | 1 | 1 | 1 | 2 | 2 | 2 | 2 | 2 | 4361 | 1 | 2 | 5 | 2 |
| 58 | 2 | 3 | N/A | 1 | 1 | 1 | 1 | 1 | 1 | 2 | 2 | 1 | 838 | 1 | 2 | 5 | 2 |
| 59 | 1 | 4 | N/A | 1 | 1 | 1 | 1 | 1 | 1 | 1 | N/A | N/A | N/A | 2 | 2 | 2 | 2 |
| 60 | 2 | 1 | N/A | 2 | 1 | 2 | 2 | 2 | 2 | 2 | N/A | N/A | N/A | 2 | 2 | 5 | 2 |
| 61 | 2 | 3 | N/A | 1 | 1 | 1 | 1 | 1 | 1 | 2 | N/A | N/A | N/A | N/A | N/A | 3 | 2 |
| 62 | 2 | 2 | N/A | 1 | 1 | 1 | 2 | 1 | 2 | 2 | N/A | N/A | N/A | N/A | N/A | 3 | 2 |
| 63 | 2 | 1 | 10 | 2 | 1 | 1 | 2 | 2 | 2 | 2 | N/A | N/A | N/A | 2 | 2 | 5 | 2 |
