# Supplementary material for: Association Between Clinical Factors and Result of Immune Checkpoint Inhibitor Related Myasthenia Gravis: A Single Center Experience and Systematic Review
Source: Front Neurol. 2022 Apr 7;13:858628. doi: 10.3389/fneur.2022.858628 (PMC9022009; doi:10.3389/fneur.2022.858628)
Supplement: Supplementary file 3 [file Table_3.DOCX]

Supplementary table 3. Quality Appraisal of the Literature Reported Cases.

| **Lead author (citation)** | **Title** | **Patient demographics** | **Current health status** | **Medical history** | **Physical exam** | **Patient disposition** | **Drug identification** | **Dosage** | **Drug reaction interface** | **Concomitant therapy** | **Adverse events** | **Discussion** |
| --- | --- | --- | --- | --- | --- | --- | --- | --- | --- | --- | --- | --- |
| Khokher et al [1] | Yes | Yes | Yes | Yes | Yes | Yes | Yes | Yes | Yes | Yes | Yes | Yes |
| Alnahhas et al [2] | Yes | Yes | Yes | Yes | Yes | Yes | Yes | Partially | Yes | Yes | Yes | Yes |
| Algaeed et al [3] | Yes | Yes | Yes | Yes | Yes | Yes | Yes | Yes | Yes | Yes | Yes | Yes |
| Derle et al [4] | Yes | Yes | Yes | Yes | Yes | Yes | Yes | Partially | Partially | Yes | Yes | Yes |
| Diamanti et al [5] | Yes | Yes | Yes | Yes | Yes | Yes | Yes | Yes | Yes | Yes | Yes | Yes |
| Fukasawa et al [6] | Yes | Yes | Yes | Yes | Yes | Yes | Yes | Partially | Yes | Yes | Yes | Yes |
| Johnson et al [7] | Yes | Yes | Yes | Yes | Yes | Yes | Yes | Yes | Yes | Yes | Yes | Yes |
| Liu et al [8] | Yes | Yes | Yes | Yes | Yes | Yes | Yes | Yes | Yes | Yes | Yes | Yes |
| Glio et al [9] | Yes | Yes | Yes | Yes | Yes | Yes | Yes | Yes | Yes | Yes | Yes | Yes |
| Liao et al [10] | Yes | Yes | Yes | Yes | Yes | Yes | Yes | Partially | Yes | Yes | Yes | Yes |
| Matthews et al [11] | Yes | Yes | Yes | Yes | Yes | Yes | Yes | Yes | Yes | Yes | Yes | Yes |
| Montes et al [12] | Yes | Yes | Yes | Yes | Yes | Yes | Yes | Yes | Yes | Yes | Yes | Partially |
| Mehta et al [13] | Yes | Yes | Yes | Yes | Yes | Yes | Yes | Partially | Yes | Yes | Yes | Yes |
| Pathak et al [14] | Yes | Yes | Yes | Yes | Yes | Yes | Yes | Yes | Yes | Yes | Yes | Yes |
| Onda et al [15] | Yes | Yes | Yes | Yes | Yes | Yes | Yes | Partially | Yes | Yes | Yes | Yes |
| Phadke et al [16] | Yes | Yes | Yes | Yes | Yes | Yes | Yes | Yes | Yes | Yes | Yes | Yes |
| Salim et al [17] | Yes | Yes | Yes | Yes | Yes | Yes | Yes | Yes | Yes | Yes | Yes | Yes |
| Shelly et al [18] | Yes | Yes | Yes | Yes | Yes | Yes | Yes | Partially | Partially | No | Yes | Yes |
| Takai et al [19] | Yes | Yes | Yes | Yes | Yes | Yes | Yes | Yes | Yes | Yes | Yes | Yes |
| Takizawa et al [20] | Yes | Yes | Yes | Yes | Yes | Yes | Yes | Yes | Yes | Yes | Yes | Yes |
| Todo et al [21] | Yes | Yes | Yes | Yes | Yes | Yes | Yes | Yes | Yes | Yes | Yes | Yes |
| Tozuka et al [22] | Yes | Yes | Yes | Yes | Yes | Yes | Yes | Yes | Yes | Yes | Yes | Partially |
| Tomisaki et al [23] | Yes | Yes | Yes | Yes | Yes | Yes | Yes | Yes | Yes | Yes | Yes | Yes |
| Wilson et al [24] | Yes | Yes | Yes | Yes | Yes | Yes | Yes | Yes | Yes | Yes | Yes | Yes |
| Zhu et al [25] | Yes | Yes | Yes | Yes | Yes | Yes | Yes | Partially | Yes | Yes | Yes | Yes |
| Dang et al [26] | Yes | Yes | Yes | Yes | Yes | Yes | Yes | Partially | Yes | Yes | Yes | Yes |
| March et al [27] | Yes | Yes | Yes | Yes | Yes | Yes | Yes | Partially | Yes | Yes | Yes | Yes |
| Rajendram et al [28] | Yes | Yes | Yes | Yes | No | Yes | Yes | Yes | Yes | Yes | Yes | Yes |
| Puwanant et al [29] | Yes | Yes | Yes | Yes | Yes | Yes | Yes | Yes | Yes | Yes | Yes | Yes |
| Shirai et al [30] | Yes | Yes | Yes | Yes | Yes | Yes | Yes | Yes | Yes | Yes | Yes | Yes |
| Yang et al [31] | Yes | Yes | Yes | Yes | Yes | Yes | Yes | Yes | Yes | Yes | Yes | Yes |
| Ziobro et al [32] | Yes | Yes | Yes | Yes | Yes | Yes | Yes | Partially | Yes | Yes | Yes | Yes |
| Jeyakumar et al [33] | Yes | Yes | Yes | Yes | Yes | Yes | Yes | Yes | Yes | Yes | Yes | Yes |
| Hayakawa et al [34] | Yes | Yes | Yes | Yes | Yes | Yes | Yes | Yes | Yes | Yes | Yes | Yes |
| Botta et al [35] | Yes | Yes | Yes | Yes | Yes | Yes | Yes | Yes | Yes | Yes | Yes | Partially |
| Safa et al [36] | Yes | Yes | Yes | Yes | Yes | Yes | Yes | Partially | Yes | Yes | Yes | Yes |
| Hibino et al [37] | Yes | Yes | Yes | Yes | Yes | Yes | Yes | Yes | Yes | Yes | Yes | Yes |
| Lau et al [38] | Yes | Yes | Yes | Yes | Yes | Yes | Yes | Yes | Yes | Yes | Yes | Yes |
| Nguyen et al [39] | Yes | Yes | Yes | Yes | Yes | Yes | Yes | Partially | Yes | Yes | Yes | Yes |
| Hajihossainlou et al [40] | Yes | Yes | Yes | Yes | Yes | Yes | Yes | Yes | Yes | Yes | Yes | Yes |

To evaluate the quality of the case reports retrieved from the literature, we used the guidelines recommended by the International Society for Pharmacoepidemiology and the International Society of Pharmacovigilance for publishing adverse events reports [41]. The assessment was carried out by one investigator and cross-checked by another investigators. We only used the items reported by the guidelines as required information, without regard to the items reported as desirable or relevant. The items appraised included: i) relevance of the title to the reported information, ii) adequate description of the patient (demographics, existing health condition, relevant past medical history, physical and laboratory abnormalities, and significant morbidity or mortality), iii) adequate description of the drug (identification of generic and trade names of the drug and the manufacturer, drug dosage, duration between drug administration and adverse events, and concomitant therapy that could potentially contributes to occurrence of adverse events), iv) adequate description of the adverse events and their outcome, and v) discussion of the evidence supporting the causal association between the drug and the adverse events. Possible item ratings are yes, partially, or no. Disagreement was resolved by discussion until consensus was reached.

[1-40]

References:

1. Khokher W, Bhuta S, Kesireddy N, Abuhelwa Z, Mhanna M, Iftikhar S, et al. Refractory Nivolumab-Induced Myasthenia Gravis Treated With Abatacept. American journal of therapeutics. 2021. Epub 2021/07/16. doi: 10.1097/mjt.0000000000001405. PubMed PMID: 34264884.

2. Alnahhas I, Wong J. A case of new-onset antibody-positive myasthenia gravis in a patient treated with pembrolizumab for melanoma. Muscle & nerve. 2017;55(6):E25-e6. Epub 2016/12/10. doi: 10.1002/mus.25496. PubMed PMID: 27935080.

3. Algaeed M, Mukharesh L, Heinzelmann M, Kaminski HJ. Pearls & Oy-sters: Pembrolizumab-induced myasthenia gravis. Neurology. 2018;91(14):e1365-e7. Epub 2018/10/03. doi: 10.1212/wnl.0000000000006278. PubMed PMID: 30275130.

4. Derle E, Benli S. Ipilimumab treatment associated with myasthenic crises and unfavorable disease course. Neurological sciences : official journal of the Italian Neurological Society and of the Italian Society of Clinical Neurophysiology. 2018;39(10):1773-4. Epub 2018/06/28. doi: 10.1007/s10072-018-3471-6. PubMed PMID: 29948466.

5. Diamanti L, Picca A, Bini P, Gastaldi M, Alfonsi E, Pichiecchio A, et al. Characterization and management of neurological adverse events during immune-checkpoint inhibitors treatment: an Italian multicentric experience. Neurological sciences : official journal of the Italian Neurological Society and of the Italian Society of Clinical Neurophysiology. 2021. Epub 2021/08/24. doi: 10.1007/s10072-021-05561-z. PubMed PMID: 34424427.

6. Fukasawa Y, Sasaki K, Natsume M, Nakashima M, Ota S, Watanabe K, et al. Nivolumab-Induced Myocarditis Concomitant with Myasthenia Gravis. Case reports in oncology. 2017;10(3):809-12. Epub 2017/10/27. doi: 10.1159/000479958. PubMed PMID: 29070994; PubMed Central PMCID: PMCPMC5649238.

7. Johnson DB, Saranga-Perry V, Lavin PJ, Burnette WB, Clark SW, Uskavitch DR, et al. Myasthenia Gravis Induced by Ipilimumab in Patients With Metastatic Melanoma. Journal of clinical oncology : official journal of the American Society of Clinical Oncology. 2015;33(33):e122-4. Epub 2014/04/30. doi: 10.1200/jco.2013.51.1683. PubMed PMID: 24778401; PubMed Central PMCID: PMCPMC4979104.

8. Liu Q, Ayyappan S, Broad A, Narita A. Pembrolizumab-associated ocular myasthenia gravis. Clinical & experimental ophthalmology. 2019;47(6):796-8. Epub 2019/03/13. doi: 10.1111/ceo.13499. PubMed PMID: 30859689.

9. Giglio D, Berntsson H, Fred Å, Ny L. Immune Checkpoint Inhibitor-Induced Polymyositis and Myasthenia Gravis with Fatal Outcome. Case reports in oncology. 2020;13(3):1252-7. Epub 2020/12/01. doi: 10.1159/000510740. PubMed PMID: 33250739; PubMed Central PMCID: PMCPMC7670383.

10. Liao B, Shroff S, Kamiya-Matsuoka C, Tummala S. Atypical neurological complications of ipilimumab therapy in patients with metastatic melanoma. Neuro-oncology. 2014;16(4):589-93. Epub 2014/02/01. doi: 10.1093/neuonc/nou001. PubMed PMID: 24482447; PubMed Central PMCID: PMCPMC3956363.

11. Mathews EP, Romito JW. Management of immune checkpoint inhibitor-related acute hypoxic neuromuscular respiratory failure using high-flow nasal cannula. Proceedings (Baylor University Medical Center). 2020;33(3):407-8. Epub 2020/07/18. doi: 10.1080/08998280.2020.1744793. PubMed PMID: 32675966; PubMed Central PMCID: PMCPMC7340452.

12. Montes V, Sousa S, Pita F, Guerreiro R, Carmona C. Myasthenia Gravis Induced by Ipilimumab in a Patient With Metastatic Melanoma. Frontiers in neurology. 2018;9:150. Epub 2018/04/19. doi: 10.3389/fneur.2018.00150. PubMed PMID: 29666602; PubMed Central PMCID: PMCPMC5891586.

13. Mehta JJ, Maloney E, Srinivasan S, Seitz P, Cannon M. Myasthenia Gravis Induced by Nivolumab: A Case Report. Cureus. 2017;9(9):e1702. Epub 2017/11/22. doi: 10.7759/cureus.1702. PubMed PMID: 29159009; PubMed Central PMCID: PMCPMC5690486.

14. Pathak R, Katel A, Massarelli E, Villaflor VM, Sun V, Salgia R. Immune Checkpoint Inhibitor-Induced Myocarditis with Myositis/Myasthenia Gravis Overlap Syndrome: A Systematic Review of Cases. The oncologist. 2021. Epub 2021/08/12. doi: 10.1002/onco.13931. PubMed PMID: 34378270.

15. Onda A, Miyagawa S, Takahashi N, Gochi M, Takagi M, Nishino I, et al. Pembrolizumab-induced Ocular Myasthenia Gravis with Anti-titin Antibody and Necrotizing Myopathy. Internal medicine (Tokyo, Japan). 2019;58(11):1635-8. Epub 2019/02/05. doi: 10.2169/internalmedicine.1956-18. PubMed PMID: 30713313; PubMed Central PMCID: PMCPMC6599941.

16. Phadke SD, Ghabour R, Swick BL, Swenson A, Milhem M, Zakharia Y. Pembrolizumab Therapy Triggering an Exacerbation of Preexisting Autoimmune Disease: A Report of 2 Patient Cases. Journal of investigative medicine high impact case reports. 2016;4(4):2324709616674316. Epub 2016/11/09. doi: 10.1177/2324709616674316. PubMed PMID: 27826593; PubMed Central PMCID: PMCPMC5084516.

17. Salim A, Tapia Rico G, Shaikh A, Brown MP. A systematic review of immune checkpoint inhibitor-related neurological adverse events and association with anti-neuronal autoantibodies. Expert opinion on biological therapy. 2021;21(9):1237-51. Epub 2021/03/02. doi: 10.1080/14712598.2021.1897101. PubMed PMID: 33645372.

18. Shelly S, Triplett JD, Pinto MV, Milone M, Diehn FE, Zekeridou A, et al. Immune checkpoint inhibitor-associated myopathy: a clinicoseropathologically distinct myopathy. Brain communications. 2020;2(2):fcaa181. Epub 2020/12/12. doi: 10.1093/braincomms/fcaa181. PubMed PMID: 33305263; PubMed Central PMCID: PMCPMC7713997.

19. Takai M, Kato D, Iinuma K, Maekawa YM, Nakane K, Tsuchiya T, et al. Simultaneous pembrolizumab-induced myasthenia gravis and myocarditis in a patient with metastatic bladder cancer: A case report. Urology case reports. 2020;31:101145. Epub 2020/03/20. doi: 10.1016/j.eucr.2020.101145. PubMed PMID: 32190548; PubMed Central PMCID: PMCPMC7068635.

20. Takizawa T, Kojima M, Suzuki S, Osada T, Kitagawa S, Nakahara J, et al. New onset of myasthenia gravis after intravesical Bacillus Calmette-Guerin: A case report and literature review. Medicine. 2017;96(46):e8757. Epub 2017/11/18. doi: 10.1097/md.0000000000008757. PubMed PMID: 29145329; PubMed Central PMCID: PMCPMC5704874 conflict of interest.

21. Todo M, Kaneko G, Shirotake S, Shimada Y, Nakano S, Okabe T, et al. Pembrolizumab-induced myasthenia gravis with myositis and presumable myocarditis in a patient with bladder cancer. IJU case reports. 2020;3(1):17-20. Epub 2020/08/04. doi: 10.1002/iju5.12128. PubMed PMID: 32743459; PubMed Central PMCID: PMCPMC7292166.

22. Tozuka T, Sugano T, Noro R, Takano N, Hisakane K, Takahashi S, et al. Pembrolizumab-induced agranulocytosis in a pulmonary pleomorphic carcinoma patient who developed interstitial lung disease and ocular myasthenia gravis. Oxford medical case reports. 2018;2018(11):omy094. Epub 2018/10/27. doi: 10.1093/omcr/omy094. PubMed PMID: 30364514; PubMed Central PMCID: PMCPMC6196766.

23. Tomisaki I. Editorial Comment to Pembrolizumab-induced myasthenia gravis with myositis and presumable myocarditis in a patient with bladder cancer. IJU case reports. 2020;3(1):20. Epub 2020/08/04. doi: 10.1002/iju5.12132. PubMed PMID: 32743460; PubMed Central PMCID: PMCPMC7292177.

24. Wilson R, Menassa DA, Davies AJ, Michael S, Hester J, Kuker W, et al. Seronegative antibody-mediated neurology after immune checkpoint inhibitors. Annals of clinical and translational neurology. 2018;5(5):640-5. Epub 2018/05/16. doi: 10.1002/acn3.547. PubMed PMID: 29761126; PubMed Central PMCID: PMCPMC5945956.

25. Zhu J, Li Y. Myasthenia gravis exacerbation associated with pembrolizumab. Muscle & nerve. 2016;54(3):506-7. Epub 2016/01/24. doi: 10.1002/mus.25055. PubMed PMID: 26802533.

26. Dang T, Macwan S, Dasanu CA. Late-onset double-seronegative myasthenia gravis syndrome and myasthenic crisis due to nivolumab use for Hodgkin's lymphoma. Journal of oncology pharmacy practice : official publication of the International Society of Oncology Pharmacy Practitioners. 2020:1078155220976797. Epub 2020/12/10. doi: 10.1177/1078155220976797. PubMed PMID: 33292071.

27. March KL, Samarin MJ, Sodhi A, Owens RE. Pembrolizumab-induced myasthenia gravis: A fatal case report. Journal of oncology pharmacy practice : official publication of the International Society of Oncology Pharmacy Practitioners. 2018;24(2):146-9. Epub 2017/02/06. doi: 10.1177/1078155216687389. PubMed PMID: 28147928.

28. Rajendram P, Torbic H, Duggal A, Campbell J, Hovden M, Dhawan V, et al. Critically ill patients with severe immune checkpoint inhibitor related neurotoxicity: A multi-center case series. Journal of critical care. 2021;65:126-32. Epub 2021/06/18. doi: 10.1016/j.jcrc.2021.05.020. PubMed PMID: 34139658.

29. Puwanant A, Isfort M, Lacomis D, Živković SA. Clinical spectrum of neuromuscular complications after immune checkpoint inhibition. Neuromuscular disorders : NMD. 2019;29(2):127-33. Epub 2019/01/15. doi: 10.1016/j.nmd.2018.11.012. PubMed PMID: 30638612.

30. Shirai T, Kiniwa Y, Sato R, Sano T, Nakamura K, Mikoshiba Y, et al. Presence of antibodies to striated muscle and acetylcholine receptor in association with occurrence of myasthenia gravis with myositis and myocarditis in a patient with melanoma treated with an anti-programmed death 1 antibody. European journal of cancer (Oxford, England : 1990). 2019;106:193-5. Epub 2018/12/12. doi: 10.1016/j.ejca.2018.10.025. PubMed PMID: 30528803.

31. Yang Y, Xu L, Wang D, Hui B, Li X, Zhou Y, et al. Anti-PD-1 and regorafenib induce severe multisystem adverse events in microsatellite stability metastatic colorectal cancer: a case report. Immunotherapy. 2021. Epub 2021/08/10. doi: 10.2217/imt-2020-0327. PubMed PMID: 34369830.

32. Ziobro AS, LaPlante RL, DeMari SR, Clark LM, Kingsley DJ, Smith AJ. Myasthenia Gravis Associated With Programmed Death-1 (PD-1) Receptor Inhibitor Pembrolizumab: A 40-day Case Report. Journal of pharmacy practice. 2021;34(1):166-70. Epub 2020/11/05. doi: 10.1177/0897190020970750. PubMed PMID: 33143531.

33. Jeyakumar N, Etchegaray M, Henry J, Lelenwa L, Zhao B, Segura A, et al. The Terrible Triad of Checkpoint Inhibition: A Case Report of Myasthenia Gravis, Myocarditis, and Myositis Induced by Cemiplimab in a Patient with Metastatic Cutaneous Squamous Cell Carcinoma. Case reports in immunology. 2020;2020:5126717. Epub 2020/07/23. doi: 10.1155/2020/5126717. PubMed PMID: 32695533; PubMed Central PMCID: PMCPMC7355354.

34. Hayakawa N, Kikuchi E, Suzuki S, Oya M. Myasthenia gravis with myositis induced by pembrolizumab therapy in a patient with metastatic urothelial carcinoma. International cancer conference journal. 2020;9(3):123-6. Epub 2020/06/26. doi: 10.1007/s13691-020-00408-4. PubMed PMID: 32582515; PubMed Central PMCID: PMCPMC7297883.

35. Botta C, Agostino RM, Dattola V, Cianci V, Calandruccio ND, Bianco G, et al. Myositis/Myasthenia after Pembrolizumab in a Bladder Cancer Patient with an Autoimmunity-Associated HLA: Immune-Biological Evaluation and Case Report. International journal of molecular sciences. 2021;22(12). Epub 2021/07/03. doi: 10.3390/ijms22126246. PubMed PMID: 34200673; PubMed Central PMCID: PMCPMC8230397.

36. Safa H, Johnson DH, Trinh VA, Rodgers TE, Lin H, Suarez-Almazor ME, et al. Immune checkpoint inhibitor related myasthenia gravis: single center experience and systematic review of the literature. Journal for immunotherapy of cancer. 2019;7(1):319. Epub 2019/11/23. doi: 10.1186/s40425-019-0774-y. PubMed PMID: 31753014; PubMed Central PMCID: PMCPMC6868691.

37. Hibino M, Maeda K, Horiuchi S, Fukuda M, Kondo T. Pembrolizumab-induced myasthenia gravis with myositis in a patient with lung cancer. Respirology case reports. 2018;6(7):e00355. Epub 2018/08/11. doi: 10.1002/rcr2.355. PubMed PMID: 30094028; PubMed Central PMCID: PMCPMC6079932.

38. Lau KH, Kumar A, Yang IH, Nowak RJ. Exacerbation of myasthenia gravis in a patient with melanoma treated with pembrolizumab. Muscle & nerve. 2016;54(1):157-61. Epub 2016/04/12. doi: 10.1002/mus.25141. PubMed PMID: 27065302.

39. Nguyen BH, Kuo J, Budiman A, Christie H, Ali S. Two cases of clinical myasthenia gravis associated with pembrolizumab use in responding melanoma patients. Melanoma research. 2017;27(2):152-4. Epub 2016/10/25. doi: 10.1097/cmr.0000000000000310. PubMed PMID: 27776019.

40. Hajihossainlou B, Vasileva A, Manthri S, Chakraborty K. Myasthenia gravis induced or exacerbated by immune checkpoint inhibitors: a rising concern. BMJ case reports. 2021;14(8). Epub 2021/08/25. doi: 10.1136/bcr-2021-243764. PubMed PMID: 34426425; PubMed Central PMCID: PMCPMC8383870.

41. Kelly WN, Arellano FM, Barnes J, Bergman U, Edwards IR, Fernandez AM, et al. Guidelines for submitting adverse event reports for publication. Pharmacoepidemiology and drug safety. 2007;16(5):581-7. Epub 2007/05/02. doi: 10.1002/pds.1399. PubMed PMID: 17471601.

[41]
